# Prevalence of atopic dermatitis in adults^☆^

**DOI:** 10.1016/j.abd.2020.10.016

**Published:** 2021-11-26

**Authors:** Maria Valeria Angles, Carolina Andrea Antonietti, Ana Clara Torre, Estefanía Juszkiewicz Franzé, Luis Daniel Mazzuoccolo, Claudio Alberto Salvador Parisi

**Affiliations:** a Department of Dermatology, Hospital Italiano de Buenos Aires, Buenos Aires, Argentina; b Adults and Pediatrics Allergy Units, Hospital Italiano de Buenos Aires, Buenos Aires, Argentina

Dear Editor,

Atopic Dermatitis (AD) is a chronic, recurrent inflammatory skin disease affecting between 15% and 20% of children, and 2% and 3% of adults.^1^ Cases with onset in childhood and persistence of the disease through adulthood (10%–30%) tend to have a history of atopy. However, when AD first presents at an adult age, there may be no such association and the diagnosis is generally more complex.^1^, ^2^

In adults, the disease has a significant impact on the quality of life, which is aggravated by the underdiagnoses and is related to a significant increase in healthcare costs.^3^ Little information is available in our country about epidemiology on AD in adults.

This study aimed to describe the prevalence of AD and its clinical features of our cohort of patients.

A cross-sectional observational study was conducted between January 1, 2015, and January 1, 2018, in adult patients that were members of a health insurance program of a Community University Hospital in Hospital Italiano, Buenos Aires (IPHI). This health insurance program covers around 160,000 members who are mainly middle-income class and inhabitants of Buenos Aires. Patient data are centrally recorded in a personal health record.

Patients older than 17 years of age, those who were active members of IPHI between January 1, 2015, and January 1, 2018, and were in follow-up for at least 6 months, were included in the study. A diagnosis of AD was defined as having 3 or more of the major Hanifin and Rajka criteria.^2^ AD was considered to be severe in the presence of at least one of the following: systemic treatment, phototherapy, complication-related hospital admissions (infections and/or skin or systemic inflammation in which active AD was the point of entry), and/or erythroderma.

Specialists in allergy and dermatology reviewed the medical records of the randomly selected patients. For robustness of the data on the features and prevalence of AD during the study period with an expected frequency of 3%,^3^ a half-width of 0%–3%, and a 95% Confidence Interval, 350 clinical records had to be evaluated. The sample size was calculated using Power and Precision software. Patients that met the inclusion criteria were selected by random sampling and the prevalence of AD over the study period was calculated using the total number of clinical records evaluated, expressed as a proportion with its confidence intervals, as the denominator.

The Institutional Ethics Committee approved the study.

Overall, 350 patients with a mean age of 60 years (SD 20) were randomly included in the study (Fig. 1); 59% (207) were female.

**Figure 1:**
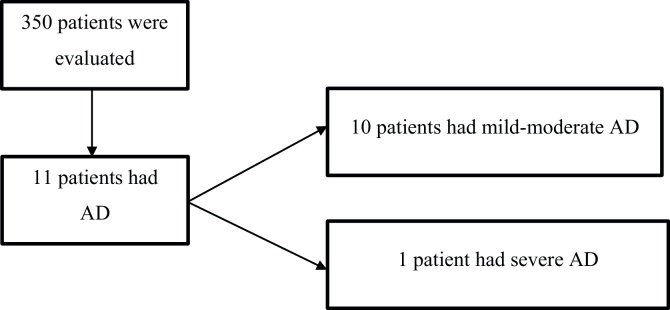
Flowchart of patients, members of a health insurance program of a Community University Hospital, evaluated in the period between January 1, 2015, and January 1, 2018.

Of the 350 patients, who were active members of the PMHI between January 1, 2015, and January 1, 2018, 11 had AD. The overall prevalence of AD was 3% (95% CI, 1.5–5.5) and the prevalence of severe AD was 0.3% (1). Of the total cohort of patients with AD, 9% (1) had severe disease. In Table 1 characteristics of the patients with AD are described.

**Table 1:** Clinical features of the patients with atopic dermatitis.

Patient-related features (%) (n = 11)	
Female sex	73% (8)
Age (years)	54 (24)^a^
Comorbidities (n = 11)	
Asthma	18% (2)
Rhinitis	36% (4)
Hypertension	9% (1)
Obesity	55% (6)
Diabetes	9% (1)
Ichthyosis vulgaris	None
Alopecia areata	18% (2)
Down’s syndrome	None
Contact dermatitis	27% (3)
Hand eczema	27% (3)
Ocular involvement (allergic conjunctivitis, keratoconus, keratoconjunctivitis)	9% (1)

AD-related features (n = 11)	
Age at diagnosis, in years	
Birth – 12 years (Childhood)	27% (3)
13–18 years (Adolescence)	9% (1)
≥19 years (Adulthood)	55% (6)
Age at diagnosis unknown	9% (1)
History of anxiety due to lack of response to treatment and/or AD-related social stigma, and/or abulia, isolation, lack of motivation and/or social integration in the context of AD.	18% (2)


a Mean (standard deviation).

None of the patients required hospital admission due to erythroderma or skin or soft-tissue infection. Table 2 shows the treatments used in this group of patients.

**Table 2:** Characteristics of the treatment received by the patients with AD.

AD treatment received for at least two months (%) (n = 11)	
Cyclosporine	9% (1)
Methotrexate	0%
UVB, and/or UVA, and/or PUVA	9% (1)
Oral methylprednisolone	18% (2)
Subcutaneous dupilumab	9% (1)
Topical treatment: humectants, emollients, corticosteroids, and/or immunomodulators	82% (9)
Mean number of consultations with an allergy specialist	0^a^
Median number of consultations with a dermatologist	2 (2–6)^b^

UVB, narrow-band ultraviolet; UVA, ultraviolet A; PUVA, psoralen plus UVA.

a Mean (standard deviation).

b Median (interquartile range).

The overall prevalence of AD in adult patients in our population was 3%. To our knowledge, this is one of the first studies exploring the prevalence of AD in Latin America. ^4^

In our study, the onset of AD was observed after the age of 19 in more than 50% of the patients. In a similar way, a recent meta-analysis published by Lee et al. reported that adulthood onset of AD was observed in 24% and 53% of the patients in Europe and America respectively.^5^

AD prevalence as well as the female preponderance (73%) in our population are in agreement with other reports.^4^ Different studies have evaluated the prevalence of AD mainly based on self-administered surveys and the Working Party criteria.^6^ A study conducted^7^ in 11 European countries and the United States, found a prevalence of AD in adults ranging from 0.3% (Switzerland) to 6.2% (Estonia), whereas other studies carried out in the United States and Japan observed a prevalence between 2.9%^4^ and 10.7%^7^ respectively. These data agree with the overall prevalence of 3% found in our study.

The study conducted in Australia^8^ is methodologically similar to ours; nevertheless, it differs in that Chidwick et al. identified patients based on AD diagnosis in the clinical records. Another difference is that in the Australian study, both adult and pediatric patients were included. The overall prevalence found in that study was two-fold higher than in ours within the population of adults older than 20 years.^8^

Regarding disease severity, we observed that 10% of the patients had severe AD. This observation is similar to that of Chidwick et al., who found that 15.6% of the study population was classified as having severe disease.^8^

Among the atopic comorbidities, allergic rhinitis (36%) was the most frequently observed such as in other studies,^4^ while obesity was the most common non-atopic comorbidity (55%). In our study, psychological disturbances were seen in 18% of the patients, a rate similar to that found in the Australian study (18.9%).^8^

The main limitation of our study is that it was conducted in a middle-class population belonging to a Community University Hospital with a high rate of elderly patients. Therefore, the results may not be translatable to other populations. Nevertheless, we believe that one of the important strengths of the study is that the data were collected from electronic records, which are a reliable and safe source, unlike the self-administered surveys. Moreover, experts in AD reviewed patients’ records.

The overall prevalence of AD in adult patients was 3%, meanwhile, for the severe forms of the disease, it was 0.3%. The most frequent comorbidities were allergic rhinitis and obesity. Availability of reliable statistical data on the prevalence of AD in adults enables visualization of the disease and its impact.

An unrestricted grant was received from Sanofi Genzyme Argentina for the development of this work.

## Financial support

None declared.

## Authors' contributions

Maria Valeria Angles: Conceived the original idea, contributed to the design and implementation of the research; contributed to the implementation of the research and the analysis of the results; discussed the results and contributed to the final manuscript; approved the final manuscript.

Carolina Andrea Antonietti: Conceived the original idea, contributed to the design and implementation of the research; contributed to the implementation of the research and the analysis of the results; discussed the results and contributed to the final manuscript; approved the final manuscript.

Ana Clara Torre: Conceived the original idea, contributed to the design and implementation of the research; contributed to the implementation of the research and the analysis of the results; discussed the results and contributed to the final manuscript; approved the final manuscript.

Estefanía Juszkiewicz Franzé: Contributed to the implementation of the research and the analysis of the results; discussed the results and contributed to the final manuscript; approved the final manuscript.

Luis Daniel Mazzuoccolo: Conceived the original idea, contributed to the design and implementation of the research; supervised the project; discussed the results, and contributed to the final manuscript; approved the final manuscript.

Claudio Alberto Salvador Parisi: Conceived the original idea, contributed to the design and implementation of the research; supervised the project; discussed the results, and contributed to the final manuscript; approved the final manuscript.

## Conflicts of interest

Angles MV, Parisi CAS, Mazzuocolo LD: Investigator and speaker for Sanofi and Abbvie.

Torre AC: Speaker for Novartis and investigator for Sanofi.

Antonietti C: Investigator for Abbvie and Sanofi.

Juszkiewicz Franzé E: Investigator for Sanofi.
